# Single cell analysis *via* mass cytometry of spontaneous intestinal perforation reveals alterations in small intestinal innate and adaptive mucosal immunity

**DOI:** 10.3389/fimmu.2023.995558

**Published:** 2023-02-07

**Authors:** Oluwabunmi Olaloye, Chino Eke, Abigail Jolteus, Liza Konnikova

**Affiliations:** ^1^ Department of Pediatrics, Yale University, New Haven, CT, United States; ^2^ Division of Neonatal and Perinatal Medicine Yale University, New Haven, CT, United States; ^3^ Department of Obstetrics, Gynecology, and Reproductive Sciences, New Haven, CT, United States; ^4^ Program in Human and Translational Immunology, Yale University, New Haven, CT, United States

**Keywords:** neonate, spontaneous intestinal perforation, NEC, newborn, mucosal immunity, T cells, neutrophils

## Abstract

**Introduction:**

Spontaneous intestinal perforation (SIP) is a poorly understood severe gastrointestinal complications of prematurity which is poorly understood. Extremely premature infants born prior to 28 weeks’ gestation develop a localized perforation of the terminal ileum during the first week of life and therapy involves surgery and cessation of enteral feeds. Little is known regardj g the impact of mucosal immune dysfunction on disease pathogenesis.

**Methods:**

We performed mass cytometry time of flight (CyTOF) of small intestinal mucosa of patients with SIP (Gestational age (GA) 24 – 27 weeks, n=8) compared to patients who had surgery for non-SIP conditions (neonatal (GA >36 weeks, n=5 ) and fetal intestine from elective terminations (GA 18-21 weeks, n=4). CyTOF analysis after stimulation of T cells with PMA/Ionomycin was also performed.

**Results:**

We noted changes in innate and adaptive mucosal immunity in SIP. SIP mucosa had an expansion of ckit+ neutrophils, an influx of naïve CD4 and CD8 T cells and a reduction of effector memory T cells. SIP T cells were characterized by reduced CCR6 and CXCR3 expression and increased interferon gamma expression after stimulation.

**Discussion:**

These findings suggest that previously unrecognized immune dysregulation is associated with SIP and should be explored in future studies.

## Introduction

In the U.S, 10% of pregnancies result in premature birth prior to 37 weeks’ gestation and complications of prematurity are a leading cause of under-five years mortality (1). Spontaneous intestinal perforation (SIP) and necrotizing enterocolitis (NEC) are gastrointestinal complications of prematurity with high morbidity, mortality and economic burden (2–5). The prevalence of SIP is between 3–8% in extremely low birth weight (birthweight < 1000g) infants in the United States (5). While the etiology of SIP remains unclear, hypoxia during delivery, defects in the muscular layer of the intestine, antenatal steroid exposure, chorioamnionitis, multiple gestation, and early indomethacin use have all been implicated (6–10). Studies on the pathogenesis of SIP are limited and have failed to provide clear insights into preventive, diagnostic and therapeutic strategies. Since the first reported case of SIP over 50 years ago, no improvements have been made in the treatment of the disorder (11). The diagnosis typically occurs in the first week of life when affected infants present with abdominal distension, with or without clinical instability, and free air in the abdomen (pneumoperitoneum) evident on radiographs (12). The current standard of care is surgical intervention (drain into abdomen or resection of compromised tissue), cessation of enteral nutrition, and a course of antibiotics (13, 14). This is the same treatment utilized when there is an intestinal perforation in NEC. Given the limited understanding of disease pathophysiology, there is a lack of specific strategies to improve outcomes in at risk infants through either the treatment or prevention of SIP.

To date, studies investigating the pathogenesis of SIP have been limited. Markers of inflammation including galectin-4 (15) and pro-inflammatory cytokines, specifically IL6, IL8, IL1β, TNFα showed modest increase in infants with SIP (16). Additionally, the impact of some pre- and postnatal exposures to indomethacin and dexamethasone which are associated with the risk of developing SIP has been evaluated in a murine model (10, 17, 18). Exposure to indomethacin and dexamethasone impacts endothelial cells and subsequent loss of smooth muscle integrity could contribute to SIP susceptibility. However, no studies to date examine the mucosal immune landscape at the time of intestinal injury in neonates with SIP.

In the past decade our understanding of fetal and neonatal immunity has evolved. Recent studies suggest that by mid-gestation, human fetuses have complex and well-developed intestinal immune systems (19–21). Consequently, defects in immune regulation or development can potentially contribute to a range of intestinal diseases in premature infants, including SIP, not thought to have an immunological origin. We have previously reported on innate immune differences in SIP affected mucosa when compared to fetal, NEC, and neonatal intestine (22). In the current manuscript, we describe innate and adaptive mucosal immune dysregulation in SIP, with an influx of ckit^+^ neutrophils and naive CD4 and CD8 T cells, a reduction in effector memory T cells and reduced expression of CCR6 and CXCR3 in CD4 and CD8 T cells. This data suggests the presence of altered innate and adaptive mucosal immunity in the human intestine at the time of SIP occurrence. Thus, potentially implicating altered mucosal immune function in the pathogenesis of SIP in extremely premature neonates.

## Methods

### Human small intestinal tissue acquisition and processing

Small intestinal tissue samples from patients were obtained from patients undergoing surgery for SIP, NEC and congenital anomalies (neonatal) with institutional review board (IRB) approval (**Table 1**). Deidentified tissue samples were obtained under a discarded specimen protocol for non-human research after approval by the University of Pittsburgh IRB (IRB# PRO17070226). Human fetal small intestine (SI) from elective terminations without any known genetic defects was obtained from the University of Pittsburgh Biospecimen core after IRB approval (IRB# PRO18010491, **Table 1**). Gestational age, sex, diagnosis, and anatomic location of tissue sample were available for each sample (**Table 1**). All samples from SIP cases were from the terminal ileum. Samples from NEC and neonatal controls were included if obtained from terminal ileum or ileum. Clinical characteristics and demographic data were not available. Intestinal tissue samples were cryopreserved in 10% dimethyl sulfoxide (DMSO) in fetal bovine serum (FBS) after they were cut into small sub-centimeter pieces per our previously published protocol (23). Samples were stored at -80°C in Mr. Frosty for 24 hours then transferred into liquid nitrogen until the time of analysis.

**Table 1:** Samples used in this study C- cytof, H- H- hematoxilin & eosin staining, S- PMA/Ionomycin stimulation.

Condition	Gestatonal age (GA, weeks)	corrected GA at surgery (weeks)	Sex	Experiment
Fetal	21	n/a	M	C, H
Fetal	21	n/a	M	C, H
Fetal	18	n/a	M	C
Fetal	21	n/a	n/a	S
Neonatal	38	45	M	C, H,S
Neonatal	Term	Term	F	C, H, S
Neonatal	Term	Term	M	C, S
Neonatal	37	37	F	C, S
Neonatal	36	37	F	H, S
SIP	24	25	F	C, S,
SIP	24	34	F	S
SIP	26	28	n/a	C, S
SIP	25	26	M	C
SIP	25	26	M	C,S
SIP	25	26	M	C
SIP	25	26	F	C
SIP	24	26	F	C
SIP	26	27	M	H
SIP	27	27	F	H
NEC	25	31	n/a	C
NEC	24	29	M	C
NEC	25	33	n/a	C
NEC	23	29	F	C
NEC	31	32	M	C
NEC	23	29	F	C
NEC	25	29	F	C
NEC	32	33	M	C
NEC	32	33	M	C
NEC	39	40	F	C
NEC	26	29	M	C
NEC	28	32	F	C
NEC	24	29	F	C

### Tissue digestion

Samples were thawed and washed in RPMI Media 1640 1X (Gibco) plus 10% FBS (Corning), 1X GlutaMax, 10mM HEPES, 1X MEM NEAA, 1mM sodium pyruvate (Gibco), 100 I.U/mL penicillin and 100 micrograms streptomycin. Tissue samples were incubated overnight at 37°C in same media as well as 100µg/mL DNase1 and 100 mg/mL collagenase A. The next day, samples were dissociated on the gentleMACS Octo Dissociator with heaters (Miltenyi Biotec, Auburn, California, U.S) per the heated human tumor protocol 1. The tissue was then filtered through a 70µM nylon mesh strainer (Sigma). Single cell suspension was then made by washing with Dulbeco’s Phosphate Buffered Solution (DPBS) without Ca^2+^ and Mg^2+^ (Sigma).

### CyTOF staining

Samples used for surface immunophenotyping with antibodies (**Table S1**) were not stimulated while samples used to assess cytokine production with cytokine panel (**Table S2**) were stimulated with 50 μg/mL phorbol 12-myristate 13-acetate (PMA) and 1 μg/mL ionomycin at 37°C and 5% CO_2_ for 4h and incubated with GolgiStop and GolgiPlug (BD Biosciences) according to the manufacturer’s instructions. We stained with rhodium (Rh103, Standard Biotools) for viability. The cells were washed with cell-staining buffer (CSB) which consists of DPBS with 0.5% bovine serum albumin (Sigma) and 0.02% sodium azide. A cocktail of antibodies tagged to heavy metals (**Table S1**) was added to the suspension. For the intracellular panel samples, the cells were washed with CSB after surface staining, incubated in FOXP3 fixation and permeabilization solution (Invitrogen). Next, cells were washed with 1X FOXP3 wash buffer and incubated in intracellular antibody cocktail (**Table S2**). Next samples were washed in CSB, fixed in 1.6% paraformaldehyde (Sigma) and kept in CSB overnight at 4°C. The following day, samples were labeled with 191Ir/193Ir DNA intercalator (Standard Biotools) and shipped overnight to the Longwood Medical Area CyTOF Core of the Dana-Farber Cancer Institute. Cells were washed in MiliQ water and beads added for normalization. Samples were analyzed on Helios cytometer (Fluidigm) at an acquisition rate of 250 events/s. Bead normalization was performed and fcs files were exported to dfci pydio cloud.

### CyTOF data analysis

**Figure 1:**
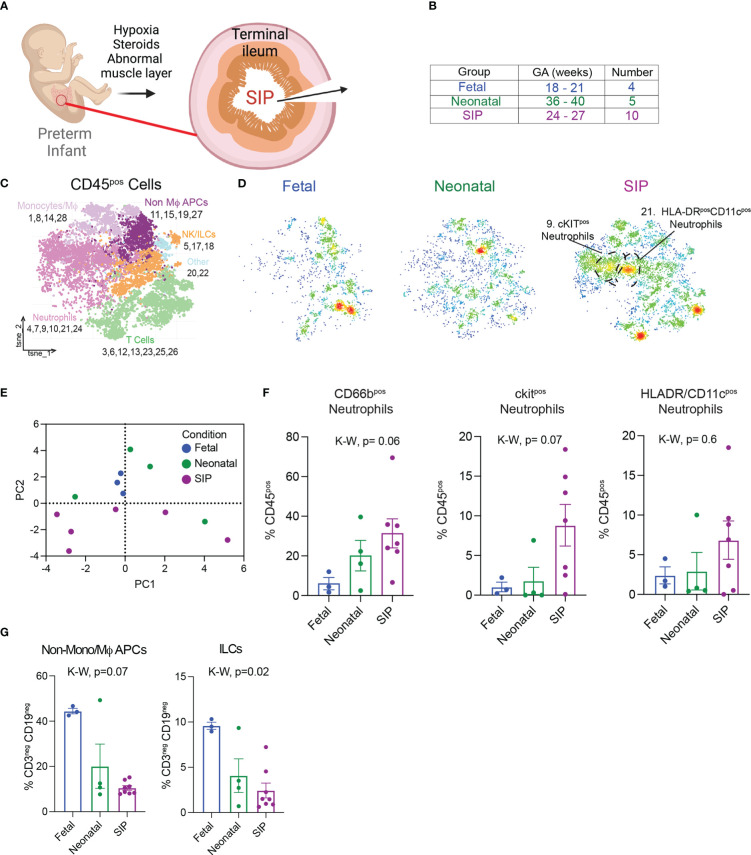
Immune landscape in neonatal spontaneous intestinal perforation **(A)** Exposures that increase susceptibility to SIP. **(B)** Samples analyzed using suspension mass cytometry (CyTOF) listed with gestational age (GA) detailed in **Table 1**. **(C).** t-stochastic neighborhood embedding (tSNE) of all leukocytes (CD45^+^) exported from Premium Cytobank after manual gating (**Figure S1A**). Populations were defined using canonical markers outlined in **Figure S1B**. **(D)** Density plots of samples in each group, fetal n=3, neonatal n=4, SIP n=8. **(E)** Principal component analysis plot based on CD45^+^ populations in C generated with GraphPad® Prism 9 (Methods). **(F)** All, ckit^+^, HLADR/CD11c^+^ neutrophils expressed as a percentage of all CD45^+^ cells. **(G)** Non-mono/MϕAPCs and ILCs expressed as a percentage of innate cells (CD3^-^CD19^-^). Each dot represents 1 case (fetal n=3, neonatal n=4, SIP n=7). *Non-Mϕ APCS – non-macrophage antigen presenting cells. NK/ILCs – natural killer/innate lymphoid cells. p-value, K-W Kruskal-Wallis test*.

**Figure 2:**
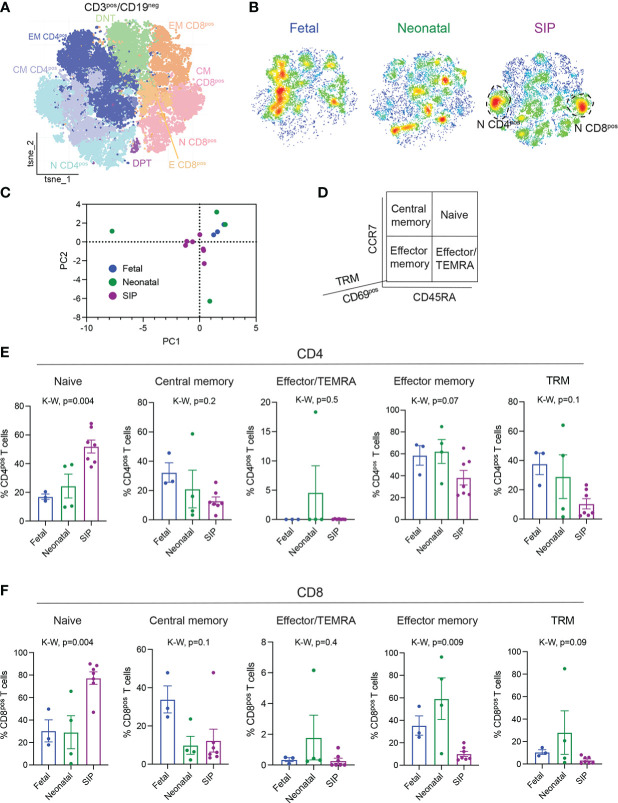
Influx of naïve CD4^+^ and CD8^+^ T cells in SIP-affected mucosa. **(A)** t-stochastic neighborhood embedding (tSNE) of all CD3^+^CD19^-^ cells. **(B)** Density plots by group fetal n=3, neonatal n=4, SIP n=7 of CD3^+^CD19^-^ cells. **(C)** Principal component analysis of CD3^+^ clusters (**Figures 2A**, **S2A**) generated in GraphPad Prism ^©^ 9 (see methods). **(D)** T cell populations are defined by CD45RA, CCR7, CD69 expression. **(E, F).** Naïve (CD45RA^+^CCR7^+^), central memory (CM, CD45RA^-^CCR7^+^), effector/TEMRA (effector memory (CD45RA^-^CCR7^-^), and tissue resident memory (TRM, CD69^+^CD45RA^-^CCR7^-^) expressed as a percentage of CD4^+^ T cells **(E)** and CD8^+^ T cells **(F**). Each dot represents 1 case (fetal n=3, neonatal n=4, SIP n=7). *p-value, K-W Kruskal-Wallis test*.

Files were uploaded to Premium Cytobank® and then gating for bead^-^, DNA^+^, Rh103^-^ and single cells^+^ was performed. The files were manually gated for CD45^+^ for all leukocytes (**Figure S1A**), CD3^-^CD19^-^CD66b^-^ for innate cells, CD3^+^ for T cells, and for intracellular cytokine analysis CD45RA^+^CCR7^+^ (naïve) and CD45RA-CCR7- (effector memory) populations were exported (**Figures S2A**, **S3A**). Next, these populations were downloaded as events from Premium Cytobank® and analyzed in cytofkit (24). Transformation in cytofkit was done with cytofAsinh, merged with ceil, and dimensionality reduction with t-Distributed Stochastic Neighbor Embedding (tSNE). Automatic clustering was performed using Rphenograph and k=30. The cluster abundances and mean metal intensity were extracted for cytofkit data in excel spreadsheets, and plots generated using GraphPad Prism®. For principal component analysis in GraphPad Prism® 9, percentages from clusters of CD45 and (PCA) were inputted as continuous variables and condition (fetal, neonatal, SIP) as a categorical variable. PCA was performed using parallel analysis with 1000 simulations and 2 components with highest variance (PC1, PC2) were selected and are displayed in **Figures 1E**, **2C**.

### Hematoxylin & eosin sections and imaging

Formalin fixed paraffin embedded sections were cut at 5 mm by the Yale Histology core and stained with hematoxylin and eosin. Slides were imaged on an Echo Revolve microscope (Echo) at 4X and 10X magnification and edited using Adobe® Illustrator.

## Results

### ckit+ neutrophils are present in SIP-affected mucosa

Although SIP is currently thought to be multifactorial (**Figure 1A**), the role of immune dysfunction has not been explored. To characterize the mucosal immune landscape at the time of injury in SIP, we reanalyzed mass cytometry data (22) from SI samples from 8 patients with SIP, 5 neonatal samples for patients who had surgery not related to NEC or SIP, and fetal intestine from elective terminations. To determine if differences noted in SIP could be due to intestinal perforationand or inflammation from surgery, we included analysis of tissue from 12 patients with NEC affecting the ileum [**Table 1**, (22)]. First, to visualize the structure of the intestinal mucosa in SIP, FFPE sections stained with H&E were imaged. In 2/3 of cases where formalin fixed paraffin embedded tissue was available, intestinal tissue structure appeared to be grossly preserved. In one case there was evidence of epithelial cell disruption (**Figure S1A**).

Next, samples (**Table S1** and **Figure 1B** **)** were analyzed using CyTOF with a panel of surface antibodies and automated unbiased clustering using RPhenograph (**Table S1**) to identify major populations including T cells, B cells, monocytes/macrophages (Mϕ), neutrophils, natural killer (NK) cells and innate lymphoid cells (ILCs, **Figures 1C**, **S2A–C** **).** The immune landscape in SIP was distinct, with a trend towards an increase in neutrophils (**Figures 1D–F**) without major differences in the abundance of most other cell types except for a relative reduction in non-monocyte/Mϕ antigen presenting cells (APCs) as well as ILCs in SIP compared to fetal and neonatal samples (**Figure 1G**). There were no differences in population of monocytes/Mϕ in SIP compared to controls (**Figure S2D**).

To identify the phenotype of neutrophilic infiltrate in SIP, the relative expression of canonical surface markers and chemokines was used (**Figure S2E**). Notably, the abundance of cluster 9 (ckit^+^ neutrophils) was significantly increased in SIP over both fetal and neonatal samples with a non-significant trend towards an increase in HLADR/CD11c^+^ neutrophils also in SIP. Ckit is a marker of immature oxidative neutrophils (**Figure S2F**). HLADR and CD11c expression on neutrophils in cluster 21, suggests that these represent a population of neutrophil/dendritic cell (DC) hybrids. Interestingly, these populations were specific to SIP and underrepresented in neonates with intestinal perforation secondary to NEC (**Figure S2E**).

### Influx of naïve and concomitant reduction in effector memory T cells in SIP

To determine if there were differences in the adaptive immunity in SIP, we explored the phenotype of the T cells present in SIP-affected mucosa. CD3^+^CD19^-^ cells were analyzed using RPhenograph clustering with identification of 29 distinct populations (**Figures 2A**, **S3A, B**). Although, we did not find abundance differences in major T cell subtypes between SIP, fetal, and neonatal tissues (**Figures S3C, D**), we again noted a unique T cell landscape in SIP compared to neonatal and fetal samples (**Figures 2B, C** **)**. There was a significant increase in naïve (CD45RA^+^CCR7^+^) CD4^+^ T cells and a decrease effector memory (EM, CD45RA^-^CCR7^-^) CD4^+^ T cells in SIP compared to fetal and neonatal SI (**Figures 2D, E** **)**. Similarly, naïve CD8^+^ T cells were increased and EM CD8^+^ T cells were reduced in SIP compared to fetal and neonatal SI (**Figure 2F**). To ensure that this phenotype is specific to SIP, we compared them to NEC samples and again noted that influx of naïve CD4^+^ and CD8^+^ T cells with a reduction in effector memory CD4^+^ T cells was unique to SIP (**Figures S4A, B**).

Tissue resident memory (TRM) T cells are a population of T cells that are present in specific tissue sites and confer local and immediate responses to infection and have been implicated in the pathogenesis of various inflammatory diseases (25–28) that are present in human fetal intestine (19–21). In SIP-affected mucosa, we identified a trend towards reduction of both CD4^+^ and CD8^+^ TRMs (CD69^+^CD45RA^-^CCR7^-^) compared to fetal SI (**Figures 2E, F** **)**, suggesting that defects in memory T cell generation could contribute to the pathogenesis of SIP. We noted a decrease in NKT cells (**Figure S5A**) and a trend towards fewer regulatory T cells (Tregs), particularly of the CD8 phenotype in SIP mucosa (**Figure S5B**).

### Altered expression of chemokines CCR6 and CXCR3 in SIP T cells

**Figure 3:**
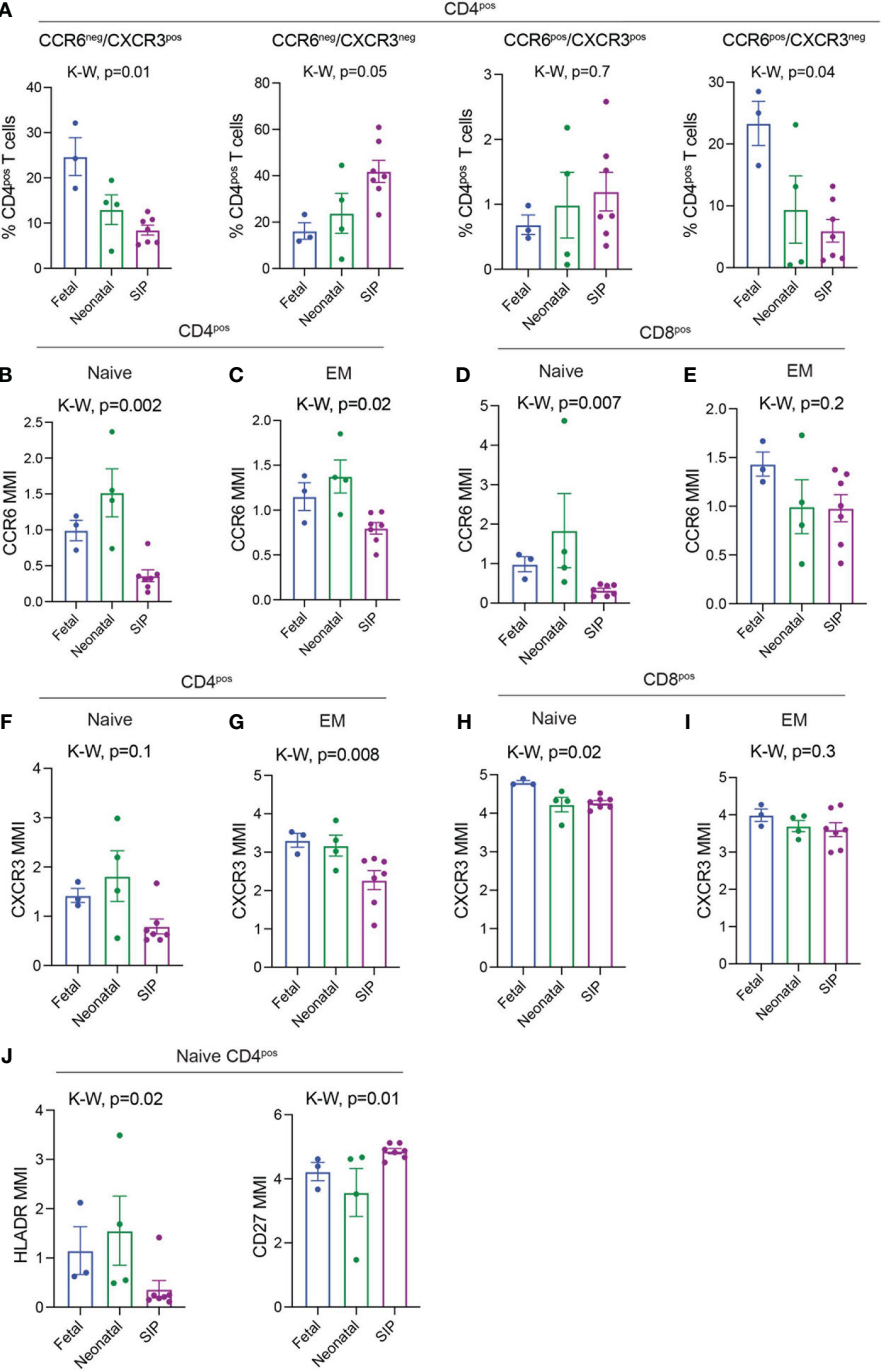
CCR6 and CXCR3 expression on T cells in SIP mucosa. **(A)** CCR6^-^CXCR3^+^, CCR6^-^CXCR3^-^, CCR6^+^CXCR3^+^ cells expressed as a percentage of CD4^+^ T cells. **B-E**. MMI of CCR6 expression on Naïve CD4^+^ **(B)**, EM CD4^+^ **(C)**, Naïve CD8**^+^ (D)**, EM CD8^+^ **(E)** T cells. **(F-I).** MMI of CXCR3 expression on Naïve CD4^+^ **(F)** EM CD4^+^ **(G)**, Naïve CD8**^+^ (H)**, EM CD8^+^ **(I)** T cells. **(J)** MMI of HLADR and CD27 on Naïve CD4^+^ T cells. In A, each dot represents 1 case. **B–I**: Each dot represents the median expression of CCR6 on that population of cells in each case (methods, fetal n=3, neonatal n=4, SIP n=7). EM, effector memory; MMI, mean metal intensity, p-value; K-W, Kruskal-Wallis test.

We then sought to evaluate the expression of chemokines involved in activation and migration on the T cells present in SIP-affected mucosa. CCR6 and CXCR3 expression can also be used to differentiate between different subtypes (Th1, Th2, Th17, Th1/17) of effector, antigen experienced, CD4^+^ T lymphocytes. We noted a reduction in CXCR3^+^CCR6^-^ (Th1-like) and CXCR3^+^CCR6^+^ (Th1/17-like) with an increase in CXCR3^-^CCR6^-^ (Th2-like) CD4^+^ T cells in SIP (**Figure 3A**) which has been described in peripheral blood of neonates (29). Likewise, overall CCR6 and CXCR3 MMI (mean metal intensity) was globally reduced on SIP CD4 T cells though a reduction in CCR6 and CXCR3 expression was noted only on naïve CD8 T cells (**Figures S5C, D**). To determine which cell subsets had altered CCR6 and CXCR3 expression we evaluated the mean metal intensity (MMI) for each individual T cell subset. We noted a significant reduction in the expression of CCR6 on naïve CD4, EM CD4 and naïve CD8 T cells in SIP compared to fetal and neonatal samples (**Figures 3B–E**). Similarly, the expression of CXCR3 was reduced in SIP compared to fetal and neonatal samples on naïve CD4, EM CD4 and naïve CD8 T cells (**Figures 3F–I**). CCR6 but not CXCR3 expression was reduced on CD4 T regs in SIP. Expression of CCR6 and CXCR3 were not altered on CD8 T regs in SIP (**Figures S5E, F**).

As both CXCR3 and CCR6 can be upregulated on activated T cells, we examined markers of T cell activation such as HLADR, CD27 and CD38 on SIP associated T cells. There were no changes in global expression of HLADR or CD38 on CD4 or CD8 T cells (**Figures S5C, D**). However, HLADR was significantly reduced on naïve CD4 T cells in SIP compared to both fetal and neonatal cases (**Figure 3J**). Meanwhile, CD27 expression was increased in SIP cases (**Figure 3J**). There was also a trend towards increased expression of other chemokines including CCR7 and CXCR5 on CD4 and CD8 cells (**Figures S5C, D**) Overall, this suggests a potential role of altered T cell migration and activation in the pathogenesis of SIP.

### Expression of IFN γ is increased in naïve T cells in SIP

**Figure 4:**
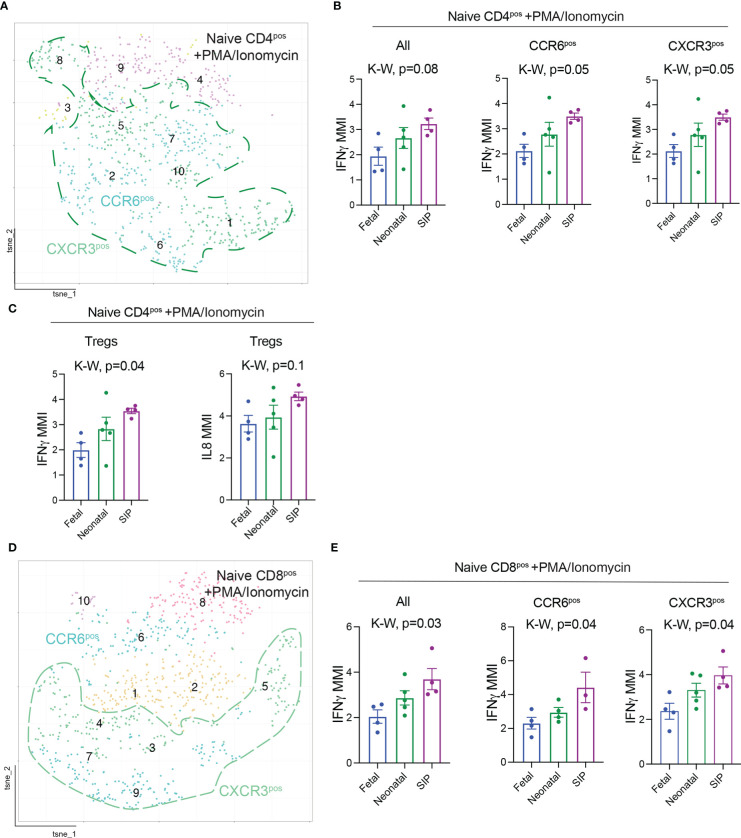
Interferon gamma (IFNγ) expression is increased in T cells in SIP. **(A)** t-SNE of naïve (CD45RA^+^CCR7^+^) CD4^+^ T cells after stimulation with PMA/Ionomycin exported from Premium Cytobank®. **(B)** MMI expression of IFNγ on all, CCR6^+^, CXCR3^+^, FOXP3^+^ naïve CD4^+^ T cells after stimulation. **(C)** MMI of IL8 on naïve T regs. **(D)** t-SNE of naïve (CD45RA^+^CCR7^+^) CD8^+^ T cells after stimulation. **(E)** MMI expression of IFNγ on all, CCR6^+^, CXCR3^+^, naïve CD8^+^ T cells after stimulation. **(B, C, E)**: Each dot represents the median expression of CCR6 on that population of cells in each case (methods, fetal n=4, neonatal n=5, SIP 4). *MMI – mean metal intensity, p-value, K-W Kruskal-Wallis test*.

To determine if cytokine expression was altered in T cells in SIP, we stimulated cells from a subset of samples (**Table 1**) with PMA/Ionomycin for non-specific T cell activation. The mean metal intensity (MMI) expression of helper T cell specific cytokines IL13, IL17A, IL21, IL22 did not differ on naïve CD4 T cells between SIP, neonatal, and fetal samples after stimulation (**Figures S6A–C**). Similarly, there was no difference in TNFα and IL1β expression among the groups. However, interferon gamma (IFNγ) expression was increased on naïve CD4^+^ and CD8^+^ T cells (**Figures 4A–E**). However, we did not see any difference in IFNγ production in EM CD4 and CD8T cells (data not shown). Additionally, IL8 expression was increased in naïve T regs in SIP compared to fetal and neonatal samples (**Figure 4C**). This data suggests a potential role of naïve T cells in promoting inflammation in the pathogenesis of SIP.

## Discussion

Spontaneous intestinal perforation is a gastrointestinal complication that affects 3-8% of extremely low birth weight infants and increases the incidence of impaired development and mortality in those affected (2, 30). Though numerous risk factors have been identified in clinical studies, mechanism specific studies of disease progression are lacking. Here, we evaluate the intestinal immune landscape in SIP-affected mucosa compared to second trimester fetal tissue, full-term neonates with congenital anomalies, and with necrotizing enterocolitis as a control with intestinal peroration. Our data reveals alterations in innate and adaptive immunity are present at the time of surgery in patients with SIP.

Neutrophils play an important role in maintaining intestinal homeostasis and are recruited to sites of active inflammation (31). They have both pro- and anti-inflammatory effects in the intestinal mucosa and are essential in the early phase of pathogen encounter and recruit other immune cells to the site of inflammation (32). Neutrophil migration to colon has been described in inflammatory bowel diseases and correlates to disease severity (33). In neonates and in animal models of necrotizing enterocolitis (NEC), neutrophils are abundant in inflamed mucosa and can exacerbate disease (22, 34–36). In SIP, we report the presence of ckit^+^ neutrophils with an immature phenotype that are essential for tissue repair (37). These could represent an early, localized response to pathogens associated with premature delivery. Neutrophilic expansion in SIP may lead to an uncontrolled inflammatory reaction due to unrestricted activation. The presence of a distinct subset of neutrophils in SIP could represent disease-specific response to local/systemic cues, activation state or acquisition of membrane proteins after immune cell interaction.

Memory T cells are the prevalent T cell subset in fetal intestine (19–21). In contrast, SIP intestine was characterized by more than 50% naïve T cells with a reduction in EM T cells, suggesting decreased memory generation *in utero* or postnatal influx of naïve T cells. Naïve T cells in SIP could be new thymic emigrants that rapidly proliferate to reconstitute the T cell pool (38). Alternatively, they could be previously accumulated naïve T cells that failed to undergo *in utero* priming. Interestingly, CD4 T cells in SIP had reduced expression of CXCR3. This is in direct contrast to the CD4 T cell profile *in utero* (20) and in newborn (21, 39) SI tissue which contains primarily CXCR3^+^ CD4 T cells. CXCR3 is a chemokine receptor that is induced on naïve T cells after activation, remains highly expressed on effector cells, plays a role in T cell migration, and facilitates the interaction of T cells with antigen presenting cells (40, 41). Decreased expression of CXCR3 suggests that there might be a defect in APC/T cell interactions that result in increased number of naïve T cells or altered migration chemokine profile of SIP associated T cells that should be further studied.

Similarly, CCR6 is crucial in the migration of T cells to the sites of inflammation (42–44) and was globally reduced in SIP associated T cells. Reduction of CCR6 and CXCR3 in SIP T cells is in contrast to increased expression of these migration chemokines that has been reported in inflammatory bowel disease (44, 45). CCR6 and its ligand CCL20 are abundantly expressed on T cells and play a chemotactic immune-modulatory role with alterations in this axis being implicated in IBD (46). CCR6 deficiency in a murine model altered the innate response *via* attenuating inflammatory response during peritonitis with lower NO production in macrophages after LPS stimulation (47). In a murine model of SIP, Gordon et al. highlight the role of nitric oxide (NO) in neonatal intestinal function (17). Exposure of deficient mice to dexamethasone and indomethacin resulted in the depletion of endothelial or inducible NOS suggesting a loss of S-nitrosylation species in smooth muscle (17). While this study highlighted a potential pathway for SIP occurrence in ELBW infants exposed to indomethacin and dexamethasone, no studies have examined this pathway *in vivo.* Depleted NO on endothelial cells and smooth muscle could contribute to SIP susceptibility. Furthermore, NO is crucial in mucosal immunity, is a known regulator of T cell differentiation (48, 49) and has been implicated in the pathogenesis of inflammatory bowel disease (50, 51). Reduction of NO isoforms in murine model of SIP combined with our report of CCR6 reduction in human SIP T cells could highlight a potential pathway for further study.

In intestinal immunity against intracellular pathogens, interferon gamma (IFNγ) is an essential cytokine involved in type II IFN responses (52). CD4 T lymphocytes express IFNγ typically after differentiation into T helper (Th1) (53). IFNγ-induction can result in improved immune surveillance and function in response to inflammation (52) or can lead to a pro-inflammatory state which is reported in IBD (54). IFNγ secretion in response to lipopolysaccharide stimulation in peripheral blood mononuclear cells is increased in neonates (55). In SIP-affected mucosa, we report increased IFNγ expression in naïve CD4^+^ and CD8^+^ T cells after stimulation with PMA/Ionomycin compared to fetal and neonatal intestine. Naïve T cells could be hyperresponsive to stimulation in SIP which could lead to a pro-inflammatory state.

This study has some limitations. Our cohort is small and available clinical data is limited to gestational age and sex. Data on antenatal and postnatal exposures that could alter intestinal immunity are lacking as patient samples were obtained in a de-identified manner. Additionally, adequately age-matched control tissue was difficult to acquire because healthy premature infants do not require surgery. An ideal control would require obtaining small intestinal tissue samples from healthy premature infants in the first week of life. However, we included samples from fetuses, neonates with congenital intestinal anomalies, and patients with NEC as controls. Furthermore, SIP typically occurs in the terminal ileum and only some of the control samples included were specifically from the terminal ileum. Another limitation is determining if changes in mucosal immunity in SIP are due to alterations in the microbiome including exposure to antibiotics. We included NEC patients who have been exposed to antibiotics and have an intestinal perforation, fetal intestine with low microbial burden and no antibiotic exposure, and neonatal intestines with exposure to microbes. Finally, CyTOF does not provide spatial information on immune cells *in situ*. Additional studies utilizing techniques like imaging mass cytometry would provide information about the location and cellular interactions contributing to SIP pathogenesis.

In summary, we observed the presence of ckit^+^ neutrophils and the expansion of naive T cells that produce IFNγ in infants with SIP. This could potentially be due to a compensatory mechanism of the intestinal mucosa to maintain homeostasis in the setting of premature delivery. On the contrary, reduced CCR6 and CXCR3 expression could suggest altered T cell migration or an inability to generate effector memory T cells that increase susceptibility to inflammation and subsequent intestinal perforation. Increased IFNγ production by naïve T cells in SIP, suggests a TCR independent cytokine production that would be interesting to evaluate in future studies. Our data provide insight into the mucosal immune landscape in neonates with SIP as well as insights into cell populations for future study into disease pathogenesis.

## Data availability statement

The raw data supporting the conclusions of this article will be made available by the authors, without undue reservation.

## Ethics statement

The studies involving human participants were reviewed and approved by University of Pittsburgh Institutional Review Board (IRB# PRO17070226, IRB# PRO18010491), Yale University Institutional Review Board. Written informed consent from the participants’ legal guardian/next of kin was not required to participate in this study in accordance with the national legislation and the institutional requirements.

## Author contributions

OO and LK conceived the study. OO was involved in sample collection processing and performed the CyTOF experiments and analysis. CE performed data analysis, imaging and editing of H&E sections. AJ performed CyTOF data analysis. LK and OO supervised the project. All authors contributed to the article and approved the submitted version.

## Supplementary material

Supplementary Table 1 Surface CyTOF antibody panel.

Supplementary Table 2 Antibody cocktail for PMA/Ionomycin stimulation CyTOF experiment.

Supplementary Figure 1
**(A).** Hematoxylin and eosin staining of Fetal (top), neonatal (middle) and SIP samples. Black bar 110 μm.

Supplementary Figure 2
**(A)**. Method for gating leukocytes (CD45^+^) in Premium Cytobank®. **(B)**. Clustergrammer (56) used to identify populations in . **(C).** Innate cells, T cells, as a percentage of all CD45^+^ cells. **(D)**. Monocytes/Mϕ, CD16^lo^, CD16^hi^, IgG^+^ CD16^hi^, CD16^hi^ monocytes expressed as a percentage of all CD45^+^ cells. **(E).** Heatmap of selected antibodies used to classify neutrophil populations. **(F).** Neutrophil clusters increased in SIP with NEC samples from (22) included for comparison. Each dot represents 1 case (fetal n=3, neonatal n=4, NEC =12, SIP n=7, ). *p-value, K-W Kruskal-Wallis test.*

Supplementary Figure 3
**(A)**. Manual gating strategy used to identify T cells (CD3^+^CD19^-^), naïve, effector memory CD4^+^ and CD8^+^ T cells exported from Premium Cytobank and used for automated clustering in cytofkit (Methods). **(B).** Clustergrammer (56) used to identify populations in **. C-D.** CD4^+^, CD8^+^ **(C)**, DNT (CD4^-^CD8^-^), DPT (CD4^+^CD8^+^) **(D)**, expressed as a percentage of all T cells. Each dot represents 1 case (fetal n=3, neonatal n=4, SIP n=7). *p-value, K-W Kruskal-Wallis test.*

Supplementary Figure 4
**(A).** Naïve CD45RA^+^CCR7^+^), effector memory (CD45RA^-^CCR7^-^), CCR6^-^CXCR3^+^ (Th1-like), CCR6^-^CXCR3^-^(Th2-like) expressed as a percentage of CD4^+^ T cells, **(B).** Naïve CD45RA^+^CCR7^+^), effector memory (CD45RA^-^CCR7^-^) expressed as a percentage of CD8^+^ T cells, samples from patients with NEC included for comparison, ) included for comparison. Each dot represents 1 case (fetal n=3, neonatal n=4, NEC n=12, SIP n=7). *p-value, K-W Kruskal-Wallis test.*

Supplementary Figure 5
**(A).** NK cells expressed as a percentage of all T cells**. (B).** Regulatory T cells (Tregs) expressed as a percentage of all T cells (left), CD4 T regs (middle), CD8 T regs (right). **(C, D)**. MMI of CCR6, CCR7, CXCR3, CXCR5, HLADR, and CD38 on CD4 T cells **(C)** and CD8 T cells **(D)**. **(E-F)**. MMI of CCR6, CXCR3 on CD4+ T regs **(E)** and CXCR3 **(F)**. Each dot Each dot represents 1 case (fetal n=3, neonatal n=4, SIP 8). *MMI- mean metal intensity. p-value, K-W Kruskal-Wallis test.*

Supplementary Figure 6
**(A).** Clustergrammer (56) with surface markers used to identify populations in **. (B, C)** MMI expression of TNFα, IL1β (B) and IL6, IL8, IL13, IL17a, IL21, IL22 (C) on naïve CD4^+^ T cells after stimulation with PMA/Ionomycin for 4 hours. **(D)**. Clustergrammer (56) with surface markers used to identify populations in **. (E, F).** MMI expression of TNFα, IL1β **(E)** and IL6, IL8, IL13, IL17a, IL21, IL22 **(F)** on naïve CD8^+^ T cells after stimulation with PMA/Ionomycin for 4 hours. *MMI- mean metal intensity. p-value, K-W Kruskal-Wallis test.*
