# Supplementary figures and images for: Single cell analysis via mass cytometry of spontaneous intestinal perforation reveals alterations in small intestinal innate and adaptive mucosal immunity
Source: Front Immunol. 2023 Feb 7;14:995558. doi: 10.3389/fimmu.2023.995558 (PMC9941693; doi:10.3389/fimmu.2023.995558)

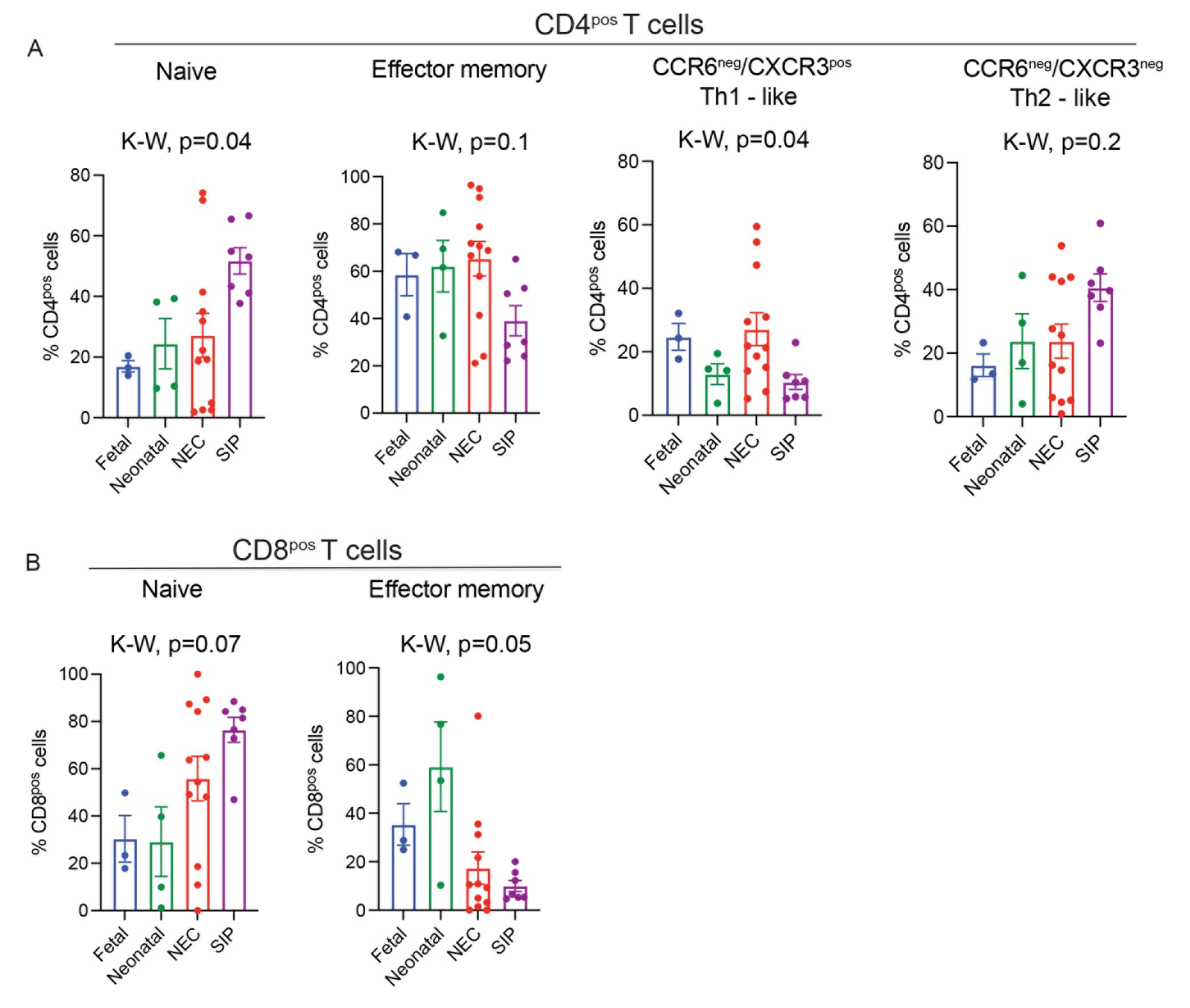

Supplement: Supplementary Figure 4 — (A). Naïve CD45RA+CCR7+), effector memory (CD45RA-CCR7-), CCR6-CXCR3+ (Th1-like), CCR6-CXCR3-(Th2-like) expressed as a percentage of CD4+ T cells, (B). Naïve CD45RA+CCR7+), effector memory (CD45RA-CCR7-) expressed as a percentage of CD8+ T cells, samples from patients with NEC included for comparison, ) included for comparison. Each dot represents 1 case (fetal n=3, neonatal n=4, NEC n=12, SIP n=7). p-value, K-W Kruskal-Wallis test. [file Image_4.tif]
